# Inference of Transcription Regulatory Network in Low Phytic Acid Soybean Seeds

**DOI:** 10.3389/fpls.2017.02029

**Published:** 2017-11-30

**Authors:** Neelam Redekar, Guillaume Pilot, Victor Raboy, Song Li, M. A. Saghai Maroof

**Affiliations:** ^1^Department of Crop and Soil Environmental Sciences, Virginia Tech, Blacksburg, VA, United States; ^2^Department of Plant Pathology, Physiology, and Weed Science, Virginia Tech, Blacksburg, VA, United States; ^3^National Small Grains Germplasm Research Center, Agricultural Research Service (USDA), Aberdeen, ID, United States

**Keywords:** phytic acid, soybean seed development, *myo*-inositol metabolism, unsupervised machine learning, gene regulatory network

## Abstract

A dominant loss of function mutation in *myo*-inositol phosphate synthase (*MIPS*) gene and recessive loss of function mutations in two multidrug resistant protein type-ABC transporter genes not only reduce the seed phytic acid levels in soybean, but also affect the pathways associated with seed development, ultimately resulting in low emergence. To understand the regulatory mechanisms and identify key genes that intervene in the seed development process in low phytic acid crops, we performed computational inference of gene regulatory networks in low and normal phytic acid soybeans using a time course transcriptomic data and multiple network inference algorithms. We identified a set of putative candidate transcription factors and their regulatory interactions with genes that have functions in myo-inositol biosynthesis, auxin-ABA signaling, and seed dormancy. We evaluated the performance of our unsupervised network inference method by comparing the predicted regulatory network with published regulatory interactions in *Arabidopsis*. Some contrasting regulatory interactions were observed in low phytic acid mutants compared to non-mutant lines. These findings provide important hypotheses on expression regulation of *myo*-inositol metabolism and phytohormone signaling in developing low phytic acid soybeans. The computational pipeline used for unsupervised network learning in this study is provided as open source software and is freely available at https://lilabatvt.github.io/LPANetwork/.

## Introduction

Seed development is a complex metabolic process, which involves both synthesis and breakdown of macromolecules for growth and maintenance of the embryo (Weber et al., 2005; Le et al., 2007). During seed development, glucose-6-phosphaste is converted to myo-inositol, an intracellular signaling molecule, which is phosphorylated several times to form phytic acid (Raboy, 1997). Seeds with reduced phytic acid content are commercially more valuable because consumption of low phytic acid seeds by monogastric animals alleviates mineral deficiency and reduces phosphorus pollution from animal waste (Raboy, 2007). Mutations that block the phytic acid biosynthesis pathway have been shown to alter the seed metabolite levels in soybean, rice, maize, and other plant species (Wilcox et al., 2000; Shi et al., 2003, 2005; Stevenson-Paulik et al., 2005; Raboy, 2007; Glover, 2011; Jervis et al., 2015). For example, a mutation in *myo-*inositol phosphate synthase (*MIPS*) gene results in reduced phytic acid, stachyose, raffinose, and elevated sucrose, and low seed emergence in soybean (Hitz et al., 2002; Saghai Maroof and Buss, 2008). Other non-biosynthetic pathway genes such as multi-drug resistance protein (*MRP*) genes encoding ATP-binding cassette transporters that are believed to be involved in the transport of phytic acid to storage vacuoles, are also known to regulate phytic acid levels and affect seed emergence (Shi et al., 2007; Nagy et al., 2009; Saghai Maroof et al., 2009; Xu et al., 2009; Jervis et al., 2015).

Transcriptome analysis is a valuable tool for the characterization of the regulatory networks that mediate this complex interaction. The expressions of genes involved in the metabolic activities in seeds are tightly regulated by the synergistic action of many transcription factors and other regulatory genes (Weber et al., 2005; Le et al., 2007). Two independent studies, one with barley low phytic acid (*lpa*) mutant, and another with soybean *mips1/mrp-l/mrp-n* (‘*3mlpa*’) triple mutant have reported the effect of *lpa* mutations on the transcriptomic profiles of developing seeds (Bowen et al., 2007; Redekar et al., 2015). The differential expression of transcription factor genes such as WRKY and CAMTA (Calmodulin-binding Transcription Activator), was linked to phytic acid biosynthesis pathway, suggesting a complex regulatory mechanism (Redekar et al., 2015). Association of WRKY transcription factors and Ca^2+^ binding activity with inositol metabolism was also confirmed by another independent study with maize low phytic acid breeding line Qi319 (Zhang et al., 2016). Zhang et al. (2016) also identified ABC transporter gene candidates associated with low phytic acid phenotype in maize using co-regulatory network. In this article, we focus on the discovery of transcription regulatory networks to further investigate the inositol metabolism in soybean.

One type of the widely applied method of network inference is the use of Pearson Correlation Coefficient or related methods for data analysis (Langfelder and Horvath, 2008; Bassel et al., 2011; Li et al., 2012). Although such correlation analyses can cluster genes with similar functions, the resultant networks do not predict the direction of gene regulation. Many other approaches, such as mutual information (Faith et al., 2007), partial correlation (Faith et al., 2007), random forest (Huynh-Thu et al., 2010), and least angle regression (LARS) (Haury et al., 2012), have been developed to perform inference of directed gene regulatory networks. For well-characterized model organisms such as *Arabidopsis*, known interactions from ChIP-chips or ChIP-seq experiments can be used as prior knowledge in supervised machine learning approaches (Maetschke et al., 2014; Ni et al., 2016). However, for biological systems where little prior information is available, such as in soybeans, unsupervised methods have to be used for network inference. In particular, three methods including co-expression analysis (Bassel et al., 2011), decision trees (Zhu et al., 2013) and mutual information (Gonzalez-Morales et al., 2016) have been successfully applied to identify functional networks in *Arabidopsis* and soybean seeds. With numerous inference methods available, it has been found that congregating the prediction results from multiple methods (so-called “community-based method”) improves the prediction accuracy as compared to any individual method (Marbach et al., 2012).

In this study, we performed computational inference of gene regulatory networks in low phytic acid mutants and the corresponding non-mutant soybean seeds from time course transcriptomic data. In addition to previously published RNA-seq data (Redekar et al., 2015), we generated new RNA-seq data of developing seeds using a pair of soybean isogenic lines, one carrying the *mips1* mutation (‘*1mlpa*’) and the other the corresponding wild type allele. We implemented a computational pipeline for unsupervised gene regulatory network inference using five different methods: ARACNE (Margolin et al., 2006), Random Forest (Huynh-Thu et al., 2010), LARS (Haury et al., 2012), partial correlation (Schafer and Strimmer, 2005b), and context likelihood relatedness (CLR) (Faith et al., 2007). To improve computational efficiency and interpretability of the inferred network, we adopted the widely used module network approach by which genes were grouped into co-expression modules and inference of gene regulation was performed between transcription factors and gene modules (Segal et al., 2003). We found that many gene modules included genes with meaningful biological functions and some gene modules showed genotype-specific expression patterns. We identified several transcription factors that were differentially expressed between developmental stages and some of the inferred regulatory interactions were specifically found in mutants or non-mutants. Genes involved in phytic acid metabolism and related metabolic processes were found in multiple modules and were predicted to be regulated by different transcription factors. For validation, the predicted interactions were compared with known regulatory interactions observed in the model plant species, *Arabidopsis thaliana*. These findings provide important hypotheses on expression regulation of *myo*-inositol metabolism, and phytohormone signaling in developing *lpa* soybeans. The computational method for inferring regulatory networks is freely available at https://lilabatvt.github.io/LPANetwork/. This method can be used to perform network inference using time series data from soybean or any other crop species.

## Materials and methods

### Genetic materials

**Figure 1:**
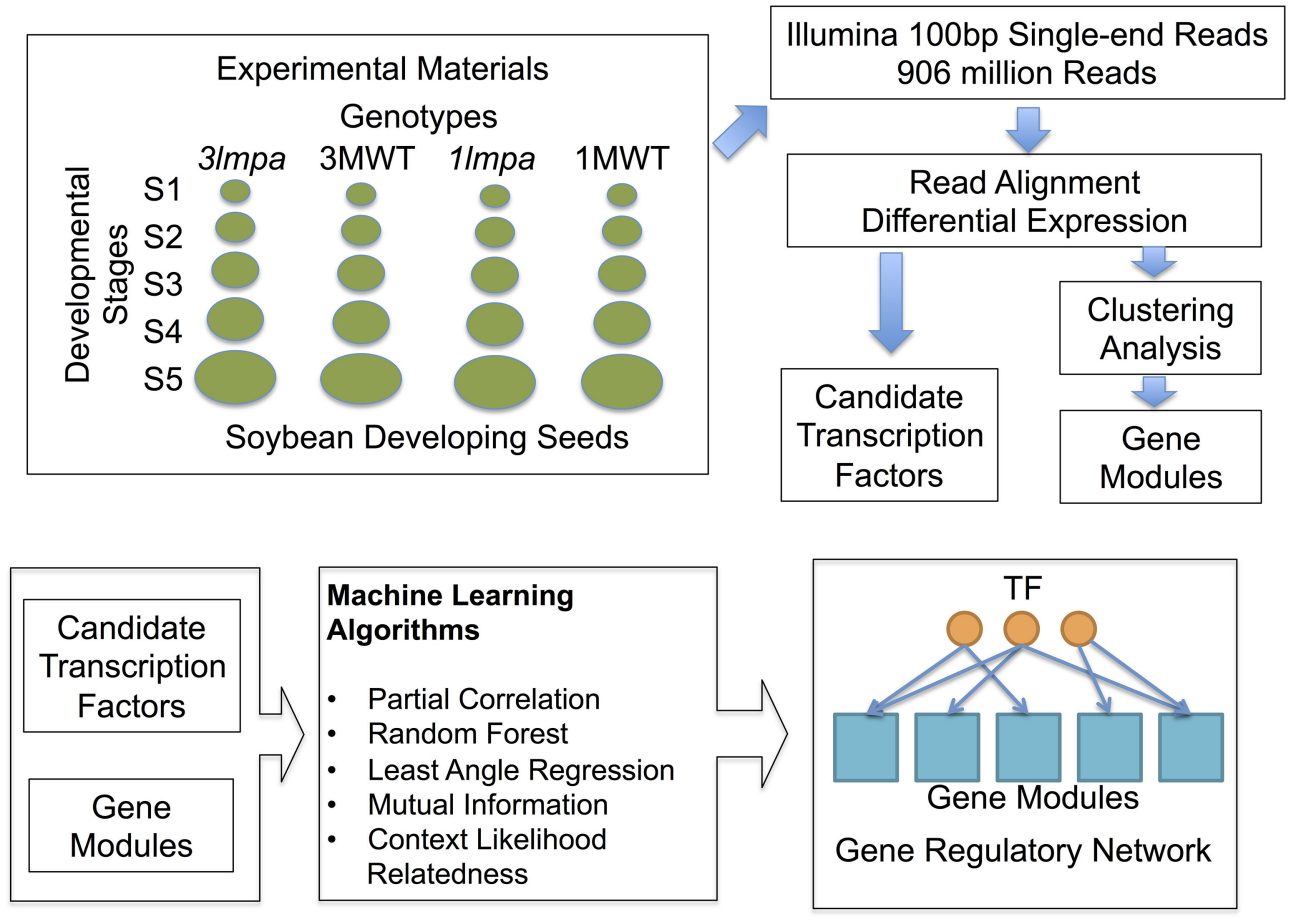
Experimental design and computational pipeline. Samples used in this study include both published (Redekar et al., 2015) and newly generated data. Differential expression analysis and clustering analysis were used to produce the initial candidate genes and gene modules. Machine learning algorithms were used to construct gene regulatory networks.

Four soybean experimental lines designated as: (i) *3mlpa*, (ii) 3MWT, (iii) *1mlpa*, and (iv) 1MWT were used in this study (Figure 1). The *lpa* mutant line, ‘*3mlpa*’, carrying three mutations *mips1/mrp-l/mrp-n*, and its non-mutant sibling line with normal phytic acid, ‘3MWT’, were derived from crossing of ‘CX-1834’ (*lpa* line with two *mpr-l/mrp-n* mutations on soybean chromosomes 19 and 3, respectively) with ‘V99-5089’ (*lpa* line with single *mips1* mutation) (Saghai Maroof et al., 2009). The low phytic acid causing mutations in the parental lines have been mapped to genes Glyma.11G238800 (MIPS1), Glyma.19g169000 (MRP-L), and Glyma.03g167800 (MRP-N) (Saghai Maroof et al., 2009). Another *lpa* line, ‘*1mlpa*’, carrying a single *mips1* mutation on soybean chromosome 11, and its isogenic sibling line with normal phytic acid, ‘1MWT’, were derived from crossing of ‘Essex’ (a normal phytic acid line with no mutations) with V99-5089 (Saghai Maroof and Buss, 2008; Glover, 2011).

### Plant growth and tissue sampling

Four seeds from each of experimental lines—*3mlpa*, 3MWT, *1mlpa*, and 1MWT, were planted in each of 12 pots containing Metro-Mix^®;^ 360 (Sun Gro) media topped with GardenPro ULTRA_LITE_ soil (Redekar et al., 2015). These plants (48/line) were grown in growth chambers with 14 /10 h photoperiod, 24°C/16°C temperature, 300–400 μE light and 50–60% relative humidity. Developing seeds were sampled in triplicates for each experimental line based on seed lengths corresponding to 2–4 mm (S1), 4–6 mm (S2), 6–8 mm (S3), 8–10 mm (S4), and 10–12 mm (S5), respectively. Samples were flash frozen using liquid nitrogen and stored at −70°C. High-quality total RNA (RIN 9–10) was extracted from frozen samples using RNeasy Plant Mini Kit, with on-column DNase digestion (QIAGEN). Total of 60 mRNA libraries were prepared from total RNA samples and sequenced as 100SE using HiSeq2000 at the Genome Quebec Innovation Center, Canada.

### Sequence data processing and differential gene expression

Reads were aligned to the latest soybean reference genome (‘Williams 82’ Wm82.a2.v1, downloaded from Phytozome^1^ with STAR (version 2.4.2) and number of reads mapped to each gene was counted using featureCount (version 1.4.6). Differential gene expression was analyzed using DESeq2 (version 1.8.2) in R (version 3.2.4). Four genotypes, *3mlpa*, 3MWT, *1mlpa*, and 1MWT, were analyzed in this data. For each pair of mutant and corresponding non-mutant, stage-wise comparison was performed to identify differentially expressed genes for each stage (Supplementary Figure 1A) (Redekar et al., 2015). For each genotype, between-stage comparisons were performed to identify differentially expressed genes between adjacent developmental stages (Supplementary Figure 1B). These analyses were performed using DESeq2 with default parameters. Between-stage comparisons and stage-wise comparisons address different type of biological question. Between-stage comparisons find genes that change between stages, but do not directly identify genes that are affected by mutations. Stage-wise comparison, on the other hand, directly finds genes that change between mutant and non-mutant lines, but does not find genes with interactions. Differentially expressed genes were the genes with FDR adjusted *p* < 0.01 and log_2_ fold change >1. Differentially expressed genes and their log_2_ fold changes are provided as Supplementary Tables 1–3. RNA-Seq data used in this study have been deposited into the NCBI Gene Expression Omnibus (GEO) repository under accession number GSE101692.

### Inference of gene regulatory networks

#### Expression clustering, gene ontology, and gene function analysis

Gene expression levels for each gene were normalized using DESeq2 and summarized as FPKM (Fragments Per Kilo-base pair per Million reads) values. The gene expression levels (FPKM values) were averaged across replicates and only differentially expressed genes were used in the clustering analysis. K-means clustering (Sherlock, 2000) was performed using R packages, and the number of clusters (K) was determined using the minimum Bayesian Information Criteria (BIC) method (Ramsey et al., 2008). In brief, K was set to be an integer number from 20 to 100 with an incremental step size of 5. For each *K*-value, k-means clustering was performed and BIC statistics were computed. The minimum BIC was achieved with *K* = 60 (Supplementary Figure 2). Gene Ontology (GO) annotation of all soybean genes was downloaded from Soybase^2^ GO enrichment analyses were performed for each gene module. Significantly enriched GO categories were selected using Fisher's exact test with FDR < 0.05 (Supplementary Table 4). Transcription factor annotation was downloaded from plant TFDB (Jin et al., 2015, 2017). Metabolic pathway genes were downloaded from the SoyCyc 7.0^3^ database from the Plant Metabolic Network^4^ website.

#### Network inference methods

To infer regulatory networks, we adopted the methods of module networks (Segal et al., 2003). First, genes were grouped into modules using the k-means clustering method. Second, differentially expressed transcription factors were used as putative regulators for network inference. In our data, we found 60 clusters (gene modules) and 1245 transcription factors that were differentially expressed in at least one comparison. The mean expression profile for each of the 60 modules was computed and the expression levels of 1245 transcription factors were included to construct an expression matrix with 1305 rows (genes) and 20 columns (five developmental stages for four experimental lines). Five distinct network inference algorithms: ARACNE (Margolin et al., 2006), Random Forest (Huynh-Thu et al., 2010), LARS (Haury et al., 2012), partial correlation (Schafer and Strimmer, 2005b), and CLR (Faith et al., 2007), were applied to this expression matrix to infer putative regulatory interactions between each transcription factor and gene modules. These methods were chosen because they represent a diverse set of computational methods for gene network inference. These methods were selected also because they were ranked as top performers in a recently published benchmark of network inference methods (DREAM challenge) (Marbach et al., 2012). Details of each method, statistical analysis of network and network validation are provided as supplementary text.

## Results

### Summary of differential gene expression analysis

**Figure 2:**
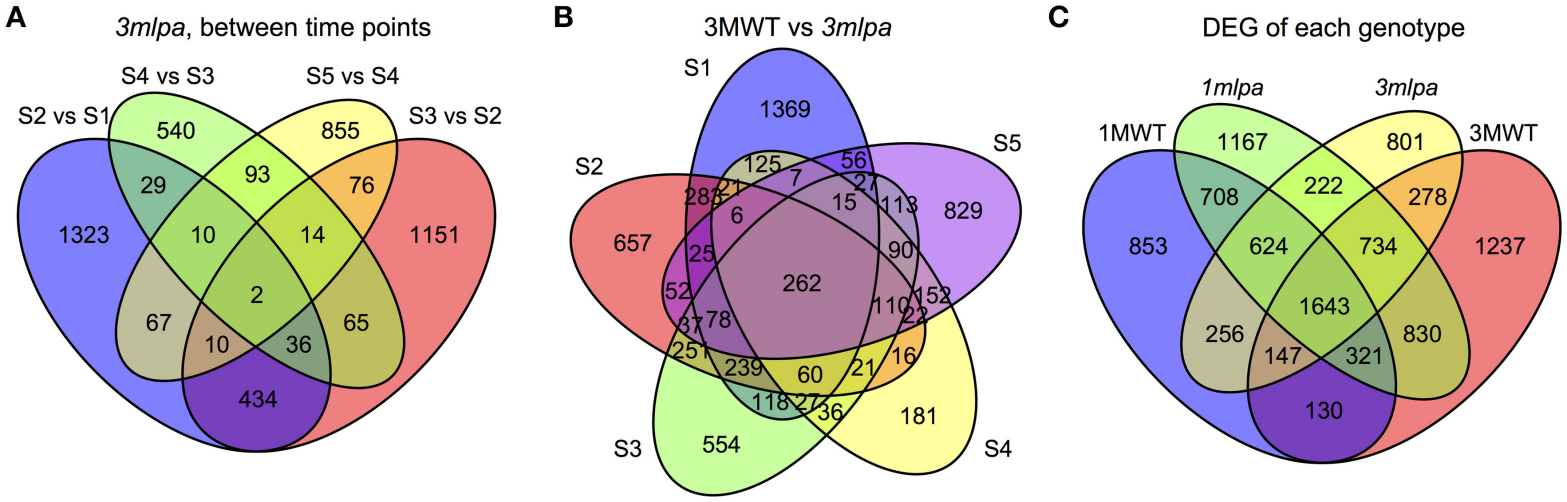
Venn diagrams. S1, S2, S3, S4, and S5 represent five developmental stages. **(A)** Number of genes differentially expressed between consecutive stages in *3mlpa* seeds. **(B)** Number of genes differentially expressed for each stage when comparing *3mlpa* to 3MWT seeds. **(C)** Number of genes differentially expressed for different genotypes for between stage comparisons.

Transcriptome sequencing data from five developing seed stages of four soybean lines (*3mlpa*, 3MWT, *1mlpa*, and 1MWT) were analyzed (Figure 1). Stage-wise comparisons were performed for mutants (*3mlpa, 1mlpa*) and their corresponding non-mutants (3MWT, 1MWT), to determine the number of genes affected by the mutation at each stage (Figure 2). For stage-wise comparisons, when *1mlpa* was compared with 1MWT, we found fewer than 250 differentially expressed genes in all time points (Supplementary Figure 2). However, we found more than 4000 differentially expressed genes between *3mlpa* and 3MWT (Supplementary Figure 2). It is expected to have higher number of differentially expressed genes for comparison between recombinant inbred lines (*3mlpa* vs. 3MWT) as opposed to that between near-isogenic lines (*1mlpa* vs. 1MWT). Few genes are differentially expressed in all five stages when comparing *3mlpa* vs. 3MWT (Figure 2B). These results suggest that genes affected by mutations are unique at each developmental stage. Between-stages comparisons were performed for each genotype separately (Figure 1, Supplementary Figure 1). Results of the stage-wise and between-stages differential expression analyses are provided as Supplementary Tables 1, 2, respectively. For between-stages comparisons (Supplementary Figure 1B), we found that hundreds of genes are differentially expressed when comparing adjacent developmental stages (Supplementary Figure 2). However, there are few genes differentially expressed across all stages. For example, in *3mlpa* mutant, only 2 genes are differentially expressed between any two adjacent stages (Figure 2A). We found 1643 genes that are differentially expressed between developmental stages and found in all four genotypes in this study (Figure 2C), suggesting these genes are a core set of genes that change expression between developmental stages and are not affected by the genotypes. We determined the number of differentially expressed genes in each of the comparisons (Supplementary Figure 2). Interestingly, the highest numbers of differentially expressed genes were found in two comparisons: between stage 1 and stage 2 for 1MWT and for 3MWT (3MWT S2 vs. S1 and 1MWT S2 vs. S1) suggesting that non-mutant plants have a high number of differentially expressed genes in the early stages of seed development than mutants.

The two sets of low-phytic acid causing mutations (*mips1* and *mrp-l/mrp-n*) interrupt the phytic acid biosynthesis and transmembrane transporter activity, ultimately reducing the seed emergence potential in our mutant lines. We were, therefore, interested in studying the behavior of genes associated with phytic acid metabolism, abscisic acid (ABA) and auxin signaling and metabolism, and transmembrane transport. The log_2_ fold change for significantly differentially expressed genes that belonged to this category is summarized in Supplementary Table 3.

### Co-expression modules represent distinct functional categories

**Figure 3:**
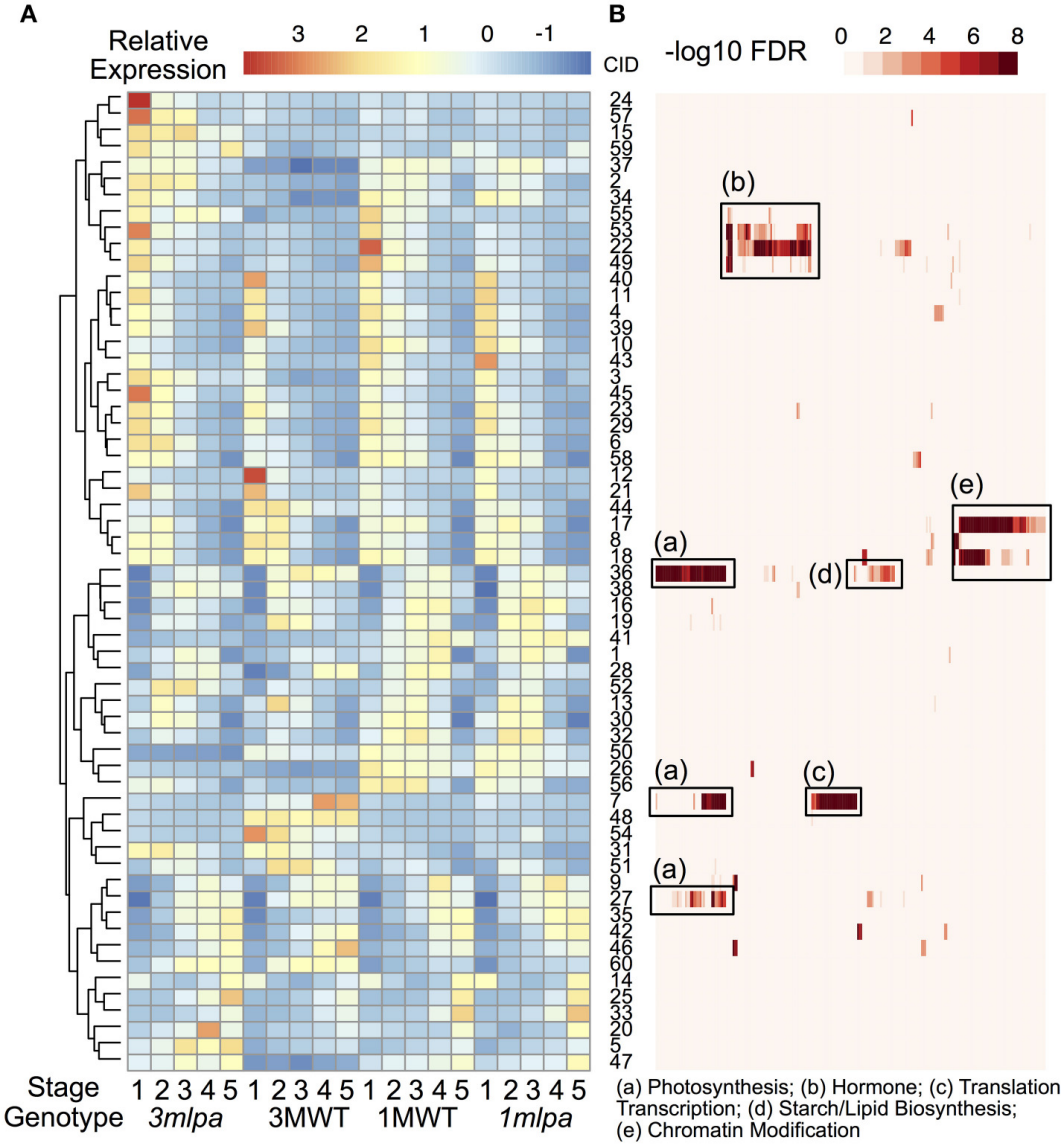
Clustering and gene function analysis. **(A)** Gene expression clusters. Color indicates normalized expression levels. Genes were clustered based on the K-means clustering algorithm. Hierarchical clustering was performed on the rows of the average expression levels for each cluster. Numbers on the right of the heatmap represent cluster id (CID, 1 to 60). **(B)** GO functional enrichment analysis. Rows of this heatmap is organized as the same order as the expression clusters. Each column represents a GO category. Color represents -log10 FDR from enrichment analysis. Some enriched categories were summarized and annotated under the heat map. The complete set of enriched GO categories is provided as supplementary information.

We identified 12998 genes that are differentially expressed in at least one of the comparisons and these genes were used for K-means clustering analysis to identify co-expressed gene modules (Figures 1, 3). We found that the optimal number of clusters (modules) is 60 based on BIC (Supplementary Figure 3). Average expression levels of all genes in each module were used to generate a heat map (Figure 3A) and GO enrichment analyses were performed for each of the modules (Figure 3B, Supplementary Table 4). The expression modules can be approximately classified into three main patterns. In pattern 1, there are 29 modules highly expressed at the early stage of seed development as shown in upper half of the heat map (Figure 3A, cluster 24 to cluster 18). In pattern 2, there are 11 modules highly expressed in the later stage of seed development as shown by the lower portion of the heat map (Figure 3A, modules 9 to 47, except for module 14, which shows high expression at both first and last developmental stages). In pattern 3, 12 modules showed high expression in the middle of the developmental stages but low expression levels in the early and late developmental stages. Genotype-specific expression patterns were also found by clustering analysis. For example, modules 24, 57, and 15 are highly expressed in *3mlpa* at the first time point, whereas the expression levels are not high in the other three genotypes. The modules identified in near isogenic lines (*1mlpa* and 1MWT) showed highly similar expression patterns than those identified in recombinant inbred lines (*3mlpa* and 3MWT).

GO enrichment analyses showed that many gene modules are enriched with genes in specific functional categories (Supplementary Table 4). For example, genes in modules 22, 49, 53, and 55 are highly expressed during the early stages of seed development and are enriched with genes with functions in hormone signaling and responses (Figure 3B, box b). Of these, several genes showed increased expression in *3mlpa* (S1) while decreased in *1mlpa* (S1) when compared with respective non-mutant lines at stage 1 (Table 1). We also found that genes in module 42 are highly expressed at the last stage of seed development and this module is enriched with genes functioning in seed dormancy, seed germination and lipid storage (Figure 3B). Genes in module 36 are highly expressed in the middle stages of seed development and are enriched with genes in starch and lipid biosynthesis (Figure 3B, box d). The functional enrichment of genes in these modules indicates that our clustering analysis can find genes representing biological functions that are known to be active at different stages of seed development. We also found that some modules have genotype-specific expression patterns. For example, module 7 is highly expressed only in 3MWT, which is enriched with genes functioning in photosynthesis, translations, and transcription (Figure 3B, boxes a,c). This result shows some gene modules have genotype-specific expression pattern and are enriched with specific functional genes.

**Table 1:** Differentially expressed genes from co-expression modules in early stages of seed development.

**Cluster ID**	**GO Term**	**Gene Name**	**Log2 fold change**		**Arabidopsis homolog**	**Gene Symbol**	**Description**
			**3mlpa vs. 3MWT-S1**	**1mlpa vs. 1MWT-S1**			
22	GO:0048838: release of seed from dormancy	Glyma.01G153300	3.13805	−2.84401	AT4G19230	*CYP707A1*	Cytochrome P450, family 707, subfamily A, polypeptide 1
	GO:0009738: abscisic acid-activated signaling pathway	Glyma.12G073000	2.67451	−2.55671	AT3G45640	*MPK3*	Mitogen-activated protein kinase 3
		Glyma.U021800	2.5946	−2.50453	AT3G45640	*MPK3*	Mitogen-activated protein kinase 3
		Glyma.07G023300	2.96832	−2.24850	AT1G80840	*WRKY40*	WRKY DNA-binding protein 40
		Glyma.10G082400	1.40473	−1.55483	AT3G11820	*SYP121, SYR1, PEN1*	Syntaxin of plants 121
		Glyma.08G271900	2.04156	−1.54559	AT1G32640	*RD22BP1, JAI1, JIN1, MYC2, ZBF1*	Basic helix-loop-helix (bHLH) DNA-binding family protein
		Glyma.03G138000	0	−1.46586	AT3G57530	*CPK32, CDPK32*	Calcium-dependent protein kinase 32
		Glyma.05G194100	0	−1.41822	AT3G52430	*PAD4*	Alpha/beta-Hydrolases superfamily protein
		Glyma.17G126400	1.18340	−1.31950	AT3G48360	*BT2*	BTB and TAZ domain protein 2
42	GO:0009845: seed germination	Glyma.17G086400	1.56707	0	AT3G01570	*-*	Oleosin family protein
49	GO:0009738: abscisic acid-activated signaling pathway	Glyma.15G078600	0	−1.84111	AT1G28480	*GRX480, roxy19*	Thioredoxin superfamily protein
		Glyma.14G066400	0	−1.10638	AT3G11410	*PP2CA, AHG3*	Protein phosphatase 2CA
53	GO:0009737: response to abscisic acid	Glyma.13G119500	1.60968	−2.02755	AT3G17510	*CIPK1, SnRK3.16*	CBL-interacting protein kinase 1
		Glyma.12G186800	2.75115	−1.95635	AT2G27310		F-box family protein
	GO:0009738: abscisic acid-activated signaling pathway	Glyma.12G186800	2.75115	−1.95635	AT2G27310		F-box family protein
	GO:0009733: response to auxin	Glyma.13G094900	4.14408	−2.26796	AT5G57560	*TCH4, XTH22*	Xyloglucan endotransglucosylase/hydrolase family protein

### Regulatory network interactions

**Figure 4:**
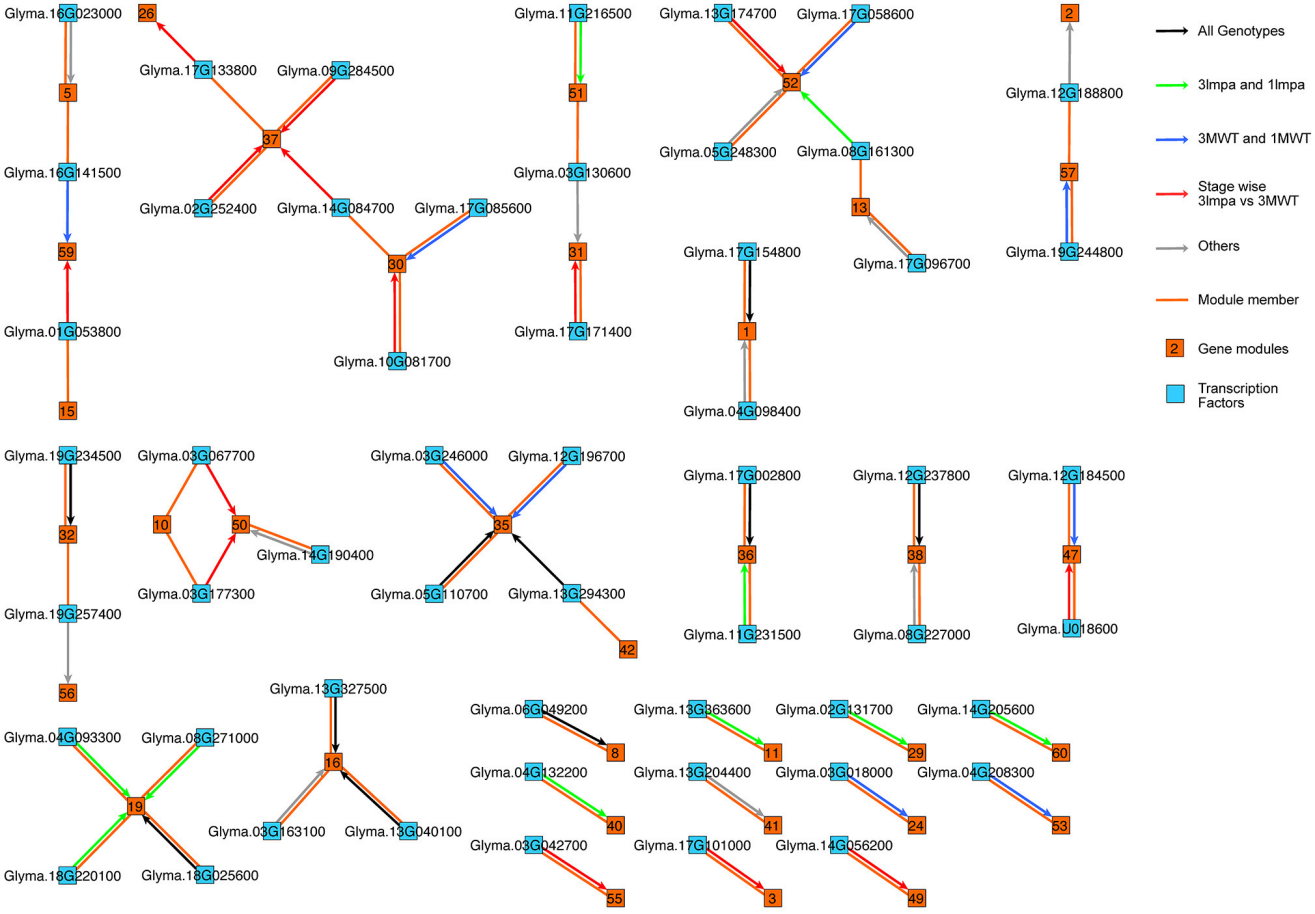
Gene regulatory networks in low phytic acid mutants and non-mutant seeds. Directed edges (with arrowheads) represent predicted regulatory interactions. Undirected edges (without arrowheads) connect each transcription factor and the co-expression module to which the transcription factor belongs. Black arrows: the TFs are differentially expressed in between stage comparisons in both mutants (*3mlpa* and *1mlpa*) and both non-mutants (3MWT and 1MWT). Green arrows: the TFs are differentially expressed in between stage comparisons for non-mutants but not in mutants. Blue arrows: the TFs are not differentially expressed in the non-mutants but are differentially expressed in either one of the mutants. Red arrows: the TFs are differentially expressed when comparing *3mlpa* to 3MWT at one or more developmental stages. Grey arrows: interactions that do not belong any of the above four categories.

Five different network inference algorithms were used to infer putative regulatory interactions between regulators and their targets (Figures 1, 4). Fifty-four interactions between 54 transcription factors and 32 modules were predicted by all five algorithms (Supplementary Table 5). Some modules were predicted to be regulated by more than one transcription factor and no transcription factor was predicted to regulate two modules. The identified interactions represent highly stringent predictions and are a very conservative estimation of all possible interactions since only 0.06% of all 74,700 possible interactions were found to be significant by all five computational methods. Four hundred six interactions between 348 transcription factors and 60 modules were supported by four or more methods (Supplementary Figure 4, Supplementary Table 6), representing a larger number for predicted regulatory interactions. The network figure includes both directed and undirected edges (Figure 4). Each directed edge connects a transcription factor with its targeted gene module. Such edge represents predicted regulatory interactions. Each undirected edge connects a transcription factor to the module this factor belongs to. These undirected edges reflect the fact that each transcription factor is also co-expressed with other genes in the genome and can be assigned to a specific gene module. The regulatory interactions (directed edges) are further classified based on the differential expression pattern of the regulatory TFs. We found 10 TF-module interactions (black arrows) in which the TFs are differentially expressed between stage comparisons (Supplementary Figure 1B) in both mutants (*3mlpa* and *1mlpa*) and both non-mutants (3MWT and 1MWT). These interactions are not affected by the mutations or genetic backgrounds. We found 10 interactions (Figure 4, green arrows) in which the TFs are differentially expressed between stage comparisons for non-mutants but not in mutants. These interactions are potentially lost due to the mutations. We also found nine interactions (blue arrows) that are not present in the non-mutants, but are present in either one of the mutants, suggesting that these interactions are gained in the mutants. Finally, we found 14 interactions (red arrows) that are not present in either the mutants or the non-mutants, but the TFs are differentially expressed when comparing *3mlpa* to 3MWT at one or more developmental stages. These interactions are altered in *3mlpa*/3MWT and do not change the trajectory of gene expression between stages but affect gene expression within specific stages. We found that many TFs are also connected to the target modules by undirected edges, indicating that these genes are co-expressed with their target genes. We also found several cases where a TF does not regulate its own module but is regulating other modules, suggesting our method can find non-linear interactions between TF and target modules. All the predicted regulatory interactions are provided as a (Supplementary Tables 5, 6).

### Validation of predicted regulatory networks

To validate the results from the computational inference, we compared the regulatory network as predicted by our methods with published regulatory interactions observed in the model plant species, *Arabidopsis*. We combined three recently published *Arabidopsis* genome-wide gene regulatory networks (Sparks et al., 2016), including 2,914 regulatory interactions between 578 regulators and 717 targets. We mapped soybean genes to *Arabidopsis* genome and searched for predicted regulator-module interactions, which are also found in *Arabidopsis*. Among 54 interactions we predicted, five TF-module interactions and 13 TF-gene interactions from this dataset are also found in the *Arabidopsis* regulatory networks (Supplementary Table 7), providing support for the predicted gene regulatory networks. This small overlapping is expected because the current *Arabidopsis* interaction network (2914 interactions) is only a tiny fraction of true interactions that happen *in-vivo* (see discussion).

Transcription factors regulate their target through binding of sequence specific motifs in the promoter regions of the target genes. To further validate the predicted regulatory networks, we performed promoter motif search. Among many programs that are available for motif discovery, the MEME suite contains the most comprehensive sets of programs that allows users to perform motif discovery, motif search and motif comparison. We used the MEME program to identify motifs in the promoter regions of genes in each gene module. We tested the enrichment of these newly discovered motifs and identified 101 motifs that are enriched in 36 modules, with each module having one or more enriched motifs. In our predicted interaction networks, there are 54 transcription factors that regulate 32 modules. We found that 21 out of these 32 modules contain one or more enriched motifs (Supplementary Table 8). These motifs are putative binding sites of the 54 transcription factors that are regulating genes in these modules.

To further validate the results of the motif search, we compared our newly discovered motifs to a database of motifs generated by direct sequencing of binding sites of over 400 *Arabidopsis* TFs. The pattern of these motifs is represented by position specific weight matrices (PSWM) (Bailey et al., 2009). This comparison aims at testing whether any of the enriched motifs are similar to the binding motifs of the genes in the same gene family in *Arabidopsis*. For example (Table 2), we found that a bZIP transcription factor (Glyma.19G244800, whose most similar *Arabidopsis* gene is AT5G28770) is regulating module 57 in our predicted network. Our analysis found a GCCACGT motif enriched in the promoter regions of module 57 (*p* < 1.21e-3). This motif is highly similar to the binding motif (GCCACGT) of an *Arabidopsis* bZIP transcription factor (*p* < 1.82e-10). Four such examples are shown in Table 2. Among the 21 modules with enriched motifs in our predicted regulatory networks, we found that 17 motifs are highly similar to the corresponding *Arabidopsis* motifs in the same TF gene family (Supplementary Table 8), providing strong support to the validity of our predictions.

**Table 2:** Motifs enrichment analysis.

**TF name**	**TF family**	**Target module name**	**ATH TF name**	**Motif enrich adj *p*-value**	**MEME motif**	**DAP-seq motif**	**Motif similarity *p*-value**
Glyma.19G244800	bZIP	57	AT5G28770	1.21E-03	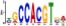	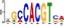	1.82E-10
Glyma.03G042700	WRKY	55	AT2G38470	1.14E-03	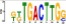	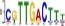	4.14E-05
Glyma.05G110700	bHLH	35	AT4G37850	6.20E-03	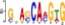	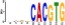	8.09E-05
Glyma.17G101000	Dof	3	AT3G47500	3.79E-04	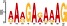	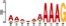	8.87E-05

“*ATH TF name” is Arabidopsis transcription factor gene homologous to the soybean transcription factor gene. “Motif enrich adj p-value” is the enrichment p-value for each of the motif found in the promoter regions of predicted gene modules. “DAP-seq motif” is the most similar motif from DAP-seq database. “Motif similarity p-value” represents the significance of similarity between the motifs found in promoters in the gene modules and the DAP-seq motifs. All significantly enriched motifs and their corresponding DAP-seq motifs are provided in Supplementary Table 8*.

### Regulatory network changes and genes in phytic acid metabolic pathways

**Figure 5:**
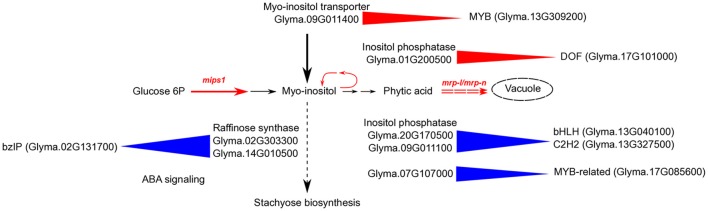
Schematic diagram of regulation of inositol pathway in low phytic acid soybean mutants. Black arrows represent the flow of *myo*-inositol in multiple pathways in non-mutant plants. Red solid arrows with *mips1* label represent mutation in the rate-limiting first step of inositol pathway, catalyzed by myo-inositol phosphate synthase. Red dashed double arrows represent mutation in MRP-type ABC transporters (*mrp-l/mrp-n*) that block the last step in the inositol pathway, which is the movement of phytic acid to storage vacuoles. The *myo*-inositol pathway is blocked in single mutant (*mips1* or *1mlpa*) at the first step, and in triple mutant (*mips1/mrp-l/mrp-n*) at both first and last steps. Blue triangles represent predicted positive regulation in non-mutants. Red triangles represent predicted gene regulations in both single and triple mutants. For example, a bZIP transcription factor (Glyma.02G131700) is homologous to the well-known ABF1, and is involved in ABA signaling. This transcription factor is predicted to positively regulate raffinose synthase in non-mutant genotypes. A DOF transcription factor (Glyma.17G101000) is predicted to regulate inositol phosphatase in mutants. This enzyme is involved in breakdown of inositol pathway intermediates to form *myo*-inositol. A MYB transcription factor (Glyma.13G309200) is predicted to regulate *myo*-inositol transporter in mutants.

To understand the connections between transcription regulation and metabolic pathways that were altered in the *3mlpa* and *1mlpa* mutant lines, we downloaded the metabolic pathway annotation from the SoyCyc 7.0 database. We mapped genes in myo-inositol metabolism, stachyose metabolism and sucrose metabolism to different gene modules, because these metabolites are altered as a result of *lpa* mutations (Supplementary Table 3). We found that there were 64 genes involved in these metabolic pathways, which mapped to 34 gene modules (Supplementary Table 3). Twelve of the 64 genes were involved in stachyose (or raffinose family oligosaccharides (RFOs)) biosynthesis and eight were associated with inferred gene regulatory networks (Figure 5). In module 29, which is up-regulated in the first stage of seed development, two genes (Glyma.02G303300 and Glyma.14G010500, both encoding raffinose synthase / seed imbibition protein 1) were found to be regulated by a bZIP transcription factor (Glyma.02G131700). This bZIP transcription factor is homologous to *ABF1* (ABA response element-binding factor 1) in *Arabidopsis*, providing a potential connection between ABA response and stachyose biosynthesis (Figure 5). Some of the enzymes involved in *myo*-inositol biosynthesis are regulated similarly in mutant and non-mutant lines. For example, inositol-polyphosphate 5-phosphatase (Glyma.20G170500) and inositol-phosphate phosphatase (Glyma.09G011100) are found in module 16. Genes in this module are up-regulated during mid-stages of seed development. Module 16 is predicted to be regulated by two transcription factors, a bHLH transcription factor (Glyma.13G040100) and a C2H2 transcription factor (Glyma.13G327500). The bHLH transcription factor is homologous to SPCH, which regulates stomatal lineage specification during embryo development (Danzer et al., 2015). Some regulatory interactions are only found in non-mutants. For example, genes from module 40 are predicted to be regulated by Glyma.17G085600, a MYB-related transcription factor, and this module contains two genes related to *myo*-inositol biosynthesis (Glyma.07G107000 encoding inositol-polyphosphate 5-phosphatase and Glyma.05G180600 encoding inositol-1-phosphate synthase). This module is highly expressed in the early stage of seed development and the MYB-related transcription factor is similar to *RSM1* in Arabidopsis, which has been found to be related to auxin signaling in early morphogenesis (Hamaguchi et al., 2008). Several target genes associated with regulation of phytic acid biosynthesis pathway matched with those discovered in co-expression network of developing maize kernel with low phytic acid content (Zhang et al., 2016). These predictions provide genotype-specific, testable hypotheses that may connect gene expression patterns with putative regulatory TFs and hormone regulations during different seed developmental stages.

## Discussion

In this study, we performed computational inference of gene regulatory networks using data from developing soybean seeds from two mutants (*3mlpa* and *1mlpa*), with *lpa*-causing mutations, and the respective non-mutant siblings (3MWT and 1MWT). We identified co-expression gene modules with distinct and genotype specific expression patterns. These gene modules are also enriched with genes with various functional categories that are related to different stages of seed development. We identified transcription factors and their putative targets using supervised machine learning methods. Some of these transcription factors are differentially expressed only in non-mutants or only in mutants, suggesting that their regulations are lost or gained due to mutations. Many genes that encode enzymes in the metabolic processes of phytic acid, myo-inositol, sucrose, and stachyose and related oligosaccharides are found in these gene modules. Overall, our analysis provides a framework to connect transcription factors with genes in biological processes such as phytic acid metabolism, auxin-abscisic acid signaling and seed dormancy.

### Importance of regulatory network inference

The predicted interactions provide a testable hypothesis for experimental validation using transgenic plants or ChIP-seq experiments. The results from this analysis can also be used to guide interpretation of other genomic mapping experiments such as genome wide association studies or quantitative trait loci analyses and to provide guidance for refining candidate gene lists. Soybean has a complex genome, which encodes over 4,500 putative transcription factors and over 46,000 coding genes. Although transcriptome data have become widely accessible for research in soybean and other crop species, it is still challenging to identify mechanistic connections between the observed transcriptome data with underlying regulatory networks. Differential gene expression analyses can be used to identify candidate genes that change under certain conditions or in specific mutants. However, in most situations, one still faces the problem of interpreting a long list of differentially expressed genes.

Our approach provides one alternative solution to this problem using well-developed machine learning methods to infer regulatory interactions. Our method implements the “community approach,” which has been shown to provide better performance than any individual method alone. The five computational methods are based on fundamentally different statistical and mathematical formulations thus complement each other and provide a list of highly confident prediction results. Our approach successfully reduces the total number of candidate genes from over 10,000 genes that change in at least one comparison to 54 transcription factors, providing a much shorter list of key genes that can be focused on for validation experiments. However, combining five methods also limited the total number of predicted interactions (54 predicted interactions), because each method predicts some interactions that are not predicted by other methods.

We validated the predicted interactions using *Arabidopsis* gene regulatory networks. *Arabidopsis* is the model plant species, which provides most gene regulatory network information among all plant species. Although soybean and *Arabidopsis* diverged over 120 million years ago, key genes in metabolic pathways and signaling networks are conserved in both species (Jung et al., 2000; Hegeman et al., 2001; Le et al., 2010; Xu et al., 2011; Leite et al., 2014; Gerrard Wheeler et al., 2016), which is expected for a physiological process as conserved as seed development. Therefore, one can expect some regulatory interactions to be conserved between these two species. In fact, 13 interactions predicted by our method are also found in the *Arabidopsis* gene regulatory networks (Supplementary Table 7). The *Arabidopsis* genome contains approximately 2200 TFs and more than 27000 genes (Jin et al., 2015, 2017; Cheng et al., 2016). There are more than 59 million possible interactions between these TF and genes. Although the number of biologically active interactions is probably < 1% of all possible interactions, the total number of true interactions is still far more than the 2914 interactions that were used in this comparison. Therefore, it is not surprising that we found a small overlap between the predicted interactions and those identified in *Arabidopsis*. As more interactions will be identified in both plant species, we would expect such overlap to increase. In our analysis, 17 motifs from 21 enriched modules (Supplementary Table 8) similar to the motifs identified in *Arabidopsis* orthologous genes, indicating that the interactions identified in this study are likely to be conserved between the two species. The actual number of conserved interactions is likely to be underestimated, because the regulatory network from *Arabidopsis* is far from complete. Nevertheless, our results provide an important first step toward characterizing gene regulatory networks in soybeans and other crop species.

One would expect more transcription factors being active during the seed development process. To observe a larger number of predicted interactions, we also provide results that are predicted by four out of five methods (Supplementary Table 6). This can be further extended to include predictions from fewer methods. Although aggregating multiple methods has been shown to outperform individual methods, some predictions by a specific method can represent interactions that cannot be detected by other approaches. If a specific target gene or specific function is of interest, one can also use our method to generate a ranked list of all predictions for the target of interest and use predicted regulators as candidate genes.

### Regulation of *myo*-inositol metabolism in *3mlpa* mutant line

*Myo-*inositol is an essential signaling molecule with multifunctional properties including gene regulation, chromatin modeling, mRNA transport, signal transduction, cell death, pathogen resistance, vesicle trafficking, plasma membrane, and cell wall formation (Martin, 1998; Stevenson et al., 2000; Chen and Xiong, 2010). It is synthesized in a two-step pathway (Figure 5), where glucose-6-phosphate is first converted to inositol monophosphate (IMP), a rate-limiting step catalyzed by MIPS1 enzyme, followed by dephosphorylation of IMP to form *myo*-inositol (Loewus and Murthy, 2000). Upon synthesis, *myo*-inositol is utilized in and recycled from multiple metabolic pathways such as biosynthesis of phytic acid and RFOs (such as stachyose and raffinose), inositol and phosphatidylinositol intermediates, auxin-inositol conjugates and glucuronic acid.

The *mips1* mutation in *Arabidopsis* is associated with reduction in cellular *myo*-inositol levels and defects in early embryogenesis (Meng et al., 2009; Chaouch and Noctor, 2010; Chen and Xiong, 2010; Donahue et al., 2010). The *mips1* mutation in soybean (as in parent V99-5089, *1mlpa* of this study) is associated with decreased levels of phytic acid and RFOs such as raffinose and stachyose, increased levels of sucrose and low emergence. The soybean *mips1* mutants also displayed normal RFO phenotype upon application of exogenous *myo-*inositol (Hitz et al., 2002). It is likely that, similar to *Arabidopsis*, the *mips1* mutation in soybean reduces *myo*-inositol levels, preventing RFO biosynthesis in parent V99-5089 (or in *1mlp*a), as *myo*-inositol is one of the starting substrates in this pathway. Since *myo*-inositol is not consumed by synthesis of RFO (it is a necessary intermediate, but recycled along the pathway), it is possible that the concentration of *myo*-inositol is reduced to such a low level such that synthesis of RFOs is greatly reduced. Sucrose is consumed in stachyose synthesis; it is therefore possible that inhibition of this pathway by the absence of *myo*-inositol is causing the increase in sucrose levels in V99-5089, due to accumulation of unused sucrose substrate.

The *mrp-l/mrp-n* mutations resulted in reduced seed phytic acid and low emergence, but did not alter the RFOs composition. In addition, the *myo-*inositol content in *mrp-l/mrp-n* mutant increases during the seed development phase prior to maturation (Israel et al., 2011). This suggests that in the presence of *mrp-l/mrp-n* mutations, the *mips1-*associated decrease in RFOs composition is restored, despite the reduced myo-inositol production due to the *mips1* mutation. This supports the prevalent hypothesis that the lack of transporters in the *mrp-l/mrp-n* mutant may trigger hydrolysis of cytoplasmic phytic acid to form inositol intermediates and *myo*-inositol, hence elevating the *myo*-inositol levels and preventing phytic acid synthesis, diverting the metabolic pathways to *myo*-inositol production (Figure 5). Another possibility is that lack of intracellular *myo*-inositol or phytic acid triggers feedback regulation that up-regulates transporters which can import inositol from the outside of the seed, hence elevating *myo*-inositol levels. This is in agreement with increase in the expression of inositol transporter genes during early stages of seed development in *3mlpa* mutant (Redekar et al., 2015).

In the present study, the inositol transporter gene (Glyma.09G011400) is also up-regulated in *1mlpa*, a single *mips1* mutant. This common up-regulation of inositol transporter in *mips1* and in *mips1/mrp-l/mrp-n* mutants suggests that these mutations are triggering the same signaling pathway (Figure 5). This inositol transporter gene belongs to module 5 and is predicted to be regulated by a MYB transcription factor (Glyma.13G309200), which represents a promising target for experimental validation (Figure 5). These target genes are in agreement with those identified in developing maize kernel with low phytic acid content (Zhang et al., 2016).

We identified over 30 genes associated with *myo*-inositol metabolism in this network analysis (Supplementary Table 3). Some of these genes belong to the modules that are only regulated in mutants. For example, an inositol-polyphosphate 5-phosphatase (Glyma.01G200500) is predicted to be regulated by a DOF transcription factor (Glyma.17G101000). This enzyme catalyzes an intermediate step in converting phytic acid to *myo*-inositol. The DOF transcription factor is differentially expressed when comparing *3mlpa* with 3MWT at stages 1, 3, and 5 and is not differentially expressed in any other comparisons. This observation is consistent with our hypothesis that in *3mlpa* mutant, phytic acid is recycled to produce *myo*-inositol, which participates in RFO synthetic pathways.

### Regulation of auxin-ABA signaling and seed dormancy-related genes in *lpa* mutants

A main goal of this work was to elucidate the molecular basis of negative downstream impacts of *lpa* mutations. Here we found one possible component of this: that in soybean these mutations greatly impact phytohormone pathways. In addition to *myo*-inositol, the importance of phytohormones such as auxin and ABA, in seed development is well-documented (Locascio et al., 2014). Auxin is a key hormone through all phases of seed development including embryogenesis, organ differentiation, endosperm formation, and seed maturation, whereas ABA is involved in onset and maintaining seed dormancy, and is active in the seed maturation and desiccation phases (Locascio et al., 2014). Cross signaling between auxin and ABA are involved in regulating seed dormancy and hence germination (Liu et al., 2013). Hormone signal transduction is controlled by regulating their biosynthesis, accumulation and distribution in different sections and stages of developing seeds by factors such as *myo*-inositol. The *Arabidopsis mips1* mutants have demonstrated defects in cotyledon development and reduced expression of basipetal auxin efflux carriers such as *PIN1* and *PIN2* (Chen and Xiong, 2010; Luo et al., 2011). The factors regulating cellular *myo*-inositol levels might therefore also regulate cross signaling of auxin and ABA. However, it is still unclear to what extent auxin-ABA cross signaling is disturbed in *lpa*-mutants.

In this study, nearly the entire spectrum of the auxin signaling pathway was identified and was differentially regulated in *1mlpa* and *3mlpa* mutants as compared to the corresponding wild types. We identified 188 auxin-related genes differentially expressed in one or more comparisons tested in this study (Supplementary Table 3). These included genes involved in auxin metabolism, transport, signal transduction, and transcription regulation. We also observed 36 genes from the ABA signaling pathway differentially expressed in one or more comparisons. The *1mlpa* vs. 1MWT comparison identified down regulation of two ABA catabolism genes in *1mlpa* (Glyma.01G153300 and Glyma.09G218600, which remove active ABA), whereas, in *3mlpa* vs. 3MWT comparison, these two genes are up-regulated.

In summary, we identified potential candidate genes that may play a role in regulating inositol metabolism, auxin-ABA signaling and seed maturation-dormancy in low phytic acid soybean during seed development. Although follow up experiments are required to validate these findings, the comprehensive regulatory network and the computational analysis pipeline of this study has set the necessary groundwork for future hypothesis driven investigations.

## Author contributions

NR and MASM designed the experiment. NR performed the sequencing experiments. NR and SL analyzed the data. SL developed the machine-learning pipeline. NR, SL, GP, VR and MASM wrote the paper.

### Conflict of interest statement

The authors declare that the research was conducted in the absence of any commercial or financial relationships that could be construed as a potential conflict of interest.

## Footnotes

^1^https://phytozome.jgi.doe.gov/

^2^https://soybase.org/

^3^http://www.plantcyc.org/databases/soycyc/7.0

^4^http://www.plantcyc.org/

## Abbreviations

*lpa*: Low phytic acid
MIPS: *myo-*inositol phosphate synthase
MRP: Multi-drug resistance protein
DEG: Differentially expressed gene
RFO: Raffinose family oligosaccharide
ABA: Abscisic acid.
