# Supplementary figures and images for: Inference of Transcription Regulatory Network in Low Phytic Acid Soybean Seeds
Source: Front Plant Sci. 2017 Nov 30;8:2029. doi: 10.3389/fpls.2017.02029 (PMC5714895; doi:10.3389/fpls.2017.02029)

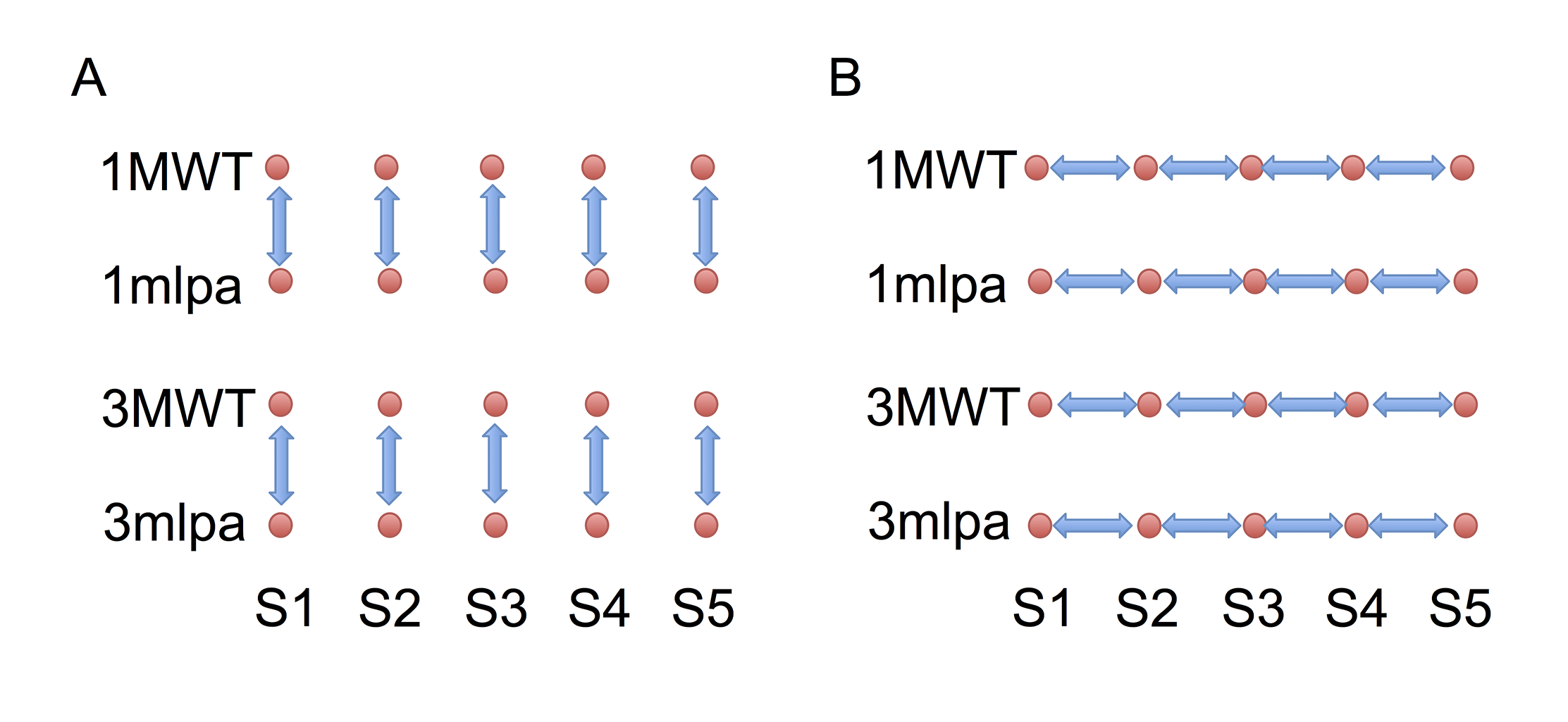

Supplement: Supplementary file 9 [file Image1.TIFF]

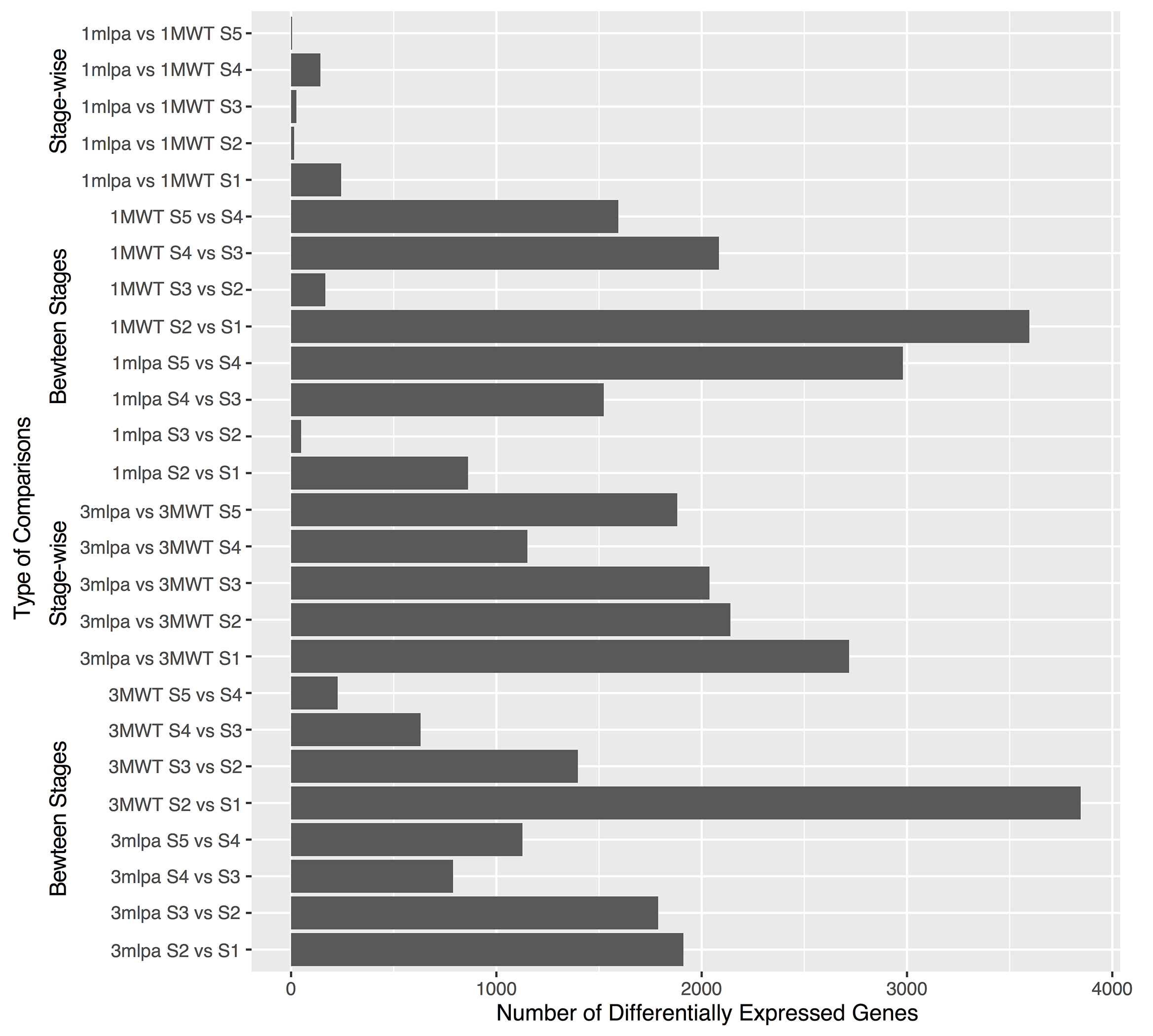

Supplement: Supplementary file 10 [file Image2.TIFF]

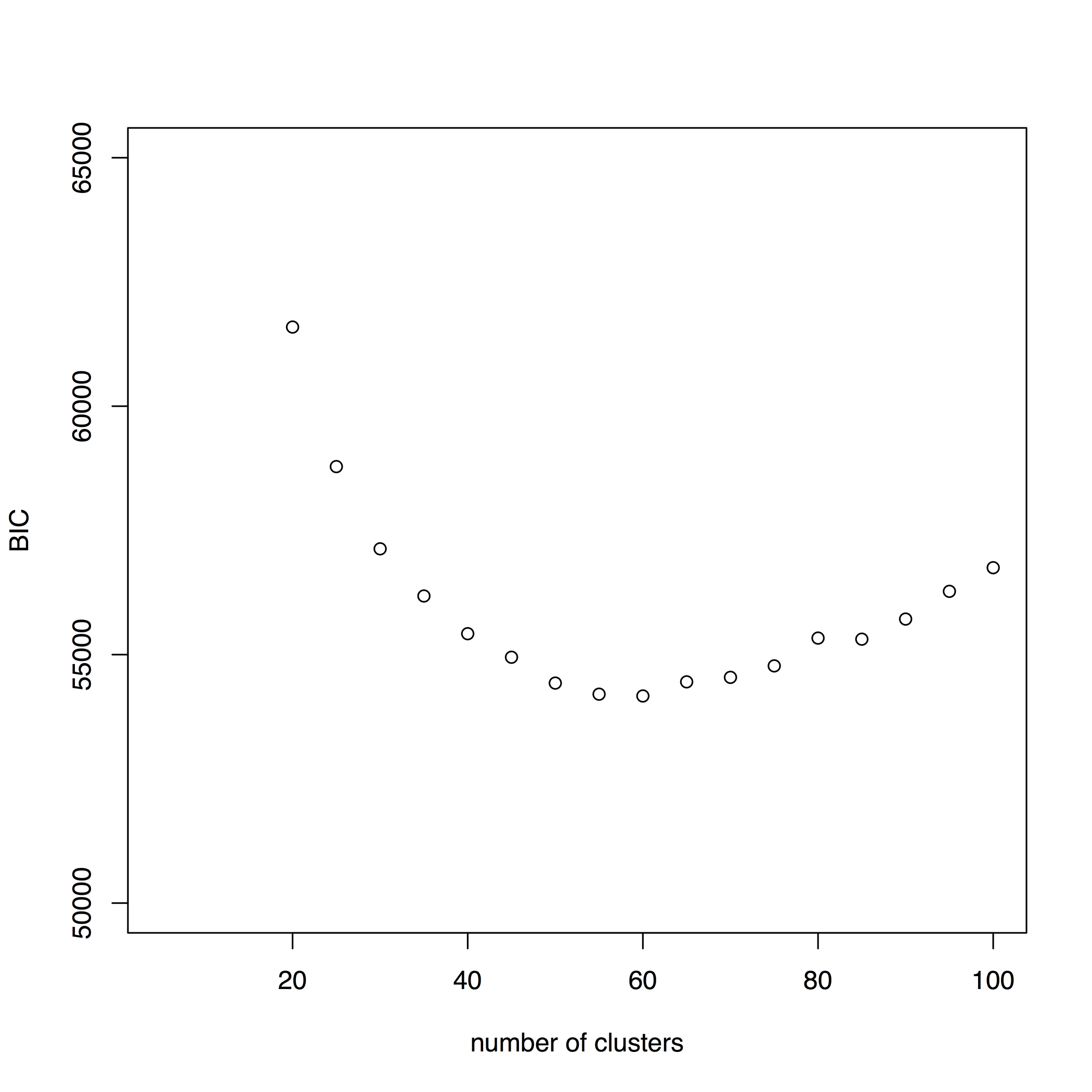

Supplement: Supplementary file 11 [file Image3.TIFF]

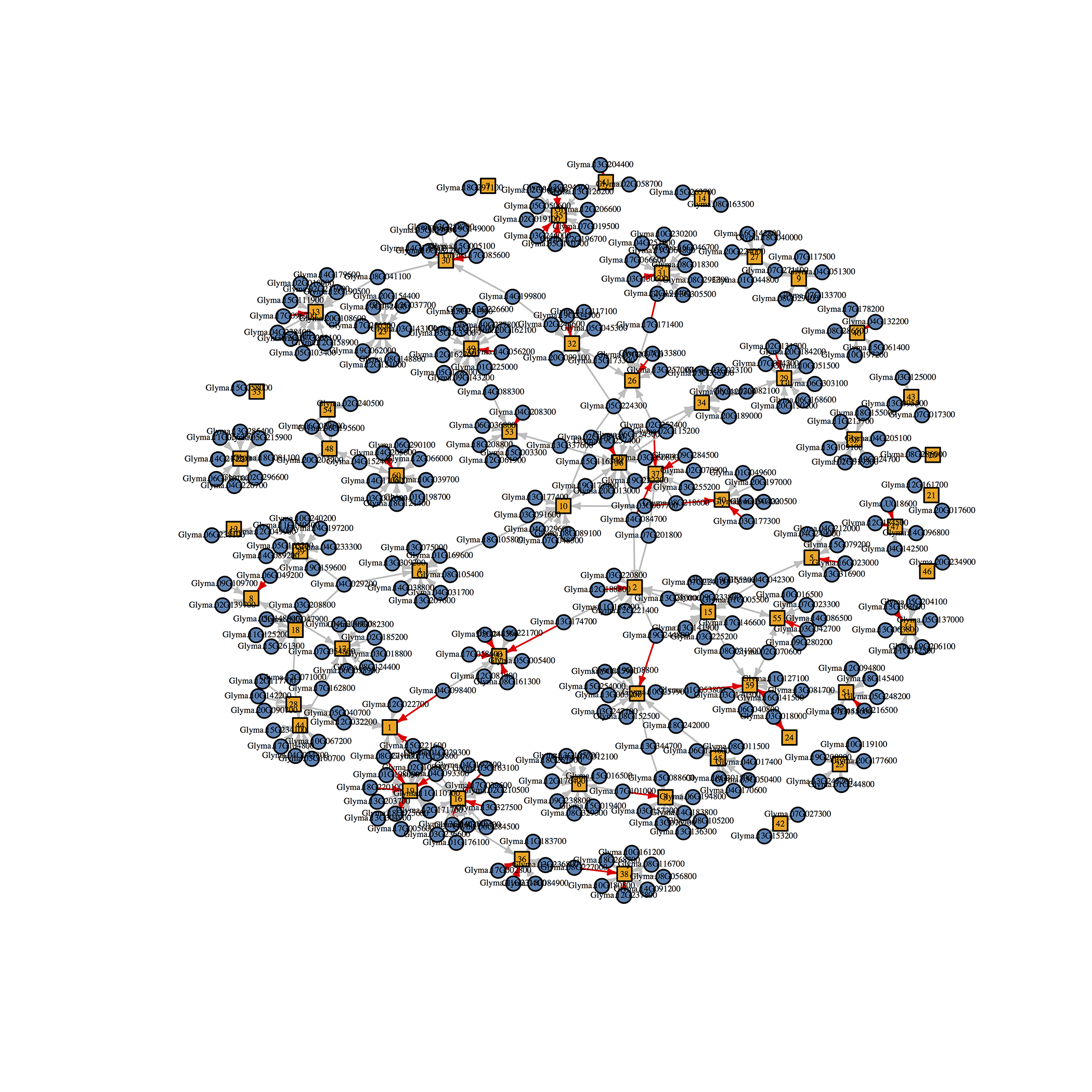

Supplement: Supplementary file 12 [file Image4.TIFF]
