# Supplementary material for: Inference of Transcription Regulatory Network in Low Phytic Acid Soybean Seeds
Source: Front Plant Sci. 2017 Nov 30;8:2029. doi: 10.3389/fpls.2017.02029 (PMC5714895; doi:10.3389/fpls.2017.02029)
Supplement: Supplementary file 13 [file DataSheet1.PDF]

# Supplementary Material

# Inference of transcription regulatory network in low phytic acid soybean seeds

**Neelam Redekar[1$], Guillaume Pilot[2], Victor Raboy[3], Song Li[1*], M. A. Saghai Maroof[1*]**
*** Correspondence:** Song Li: songli@vt.edu; M. A. Saghai Maroof: smaroof@vt.edu

## 1 Supplementary Information

### 1.1 Network inference methods

#### 1.1.1 ARACNE

ARACNE is based on mutual information, which captures dependencies between data sets that cannot be found by simple correlation analysis. The mutual information (*MI*) between a TF and the respective target module is calculated using the following equation:

$$MI(\{x_i\},\{y_i\}) = \frac{1}{M}\sum_i \frac{f(x_i,y_i)}{f(x_i) * f(y_i)}$$

In this equation, the mutual information between expressions of TFs is represented by $\{x_i\}$, and the expression of the target module is represented by $\{y_i\}$ (Margolin et al., 2006). Function $f(x_i,y_i)$ represents the joint distribution of the two random variables, whereas functions $f(x_i)$ and $f(y_i)$ represent the marginal distributions of $x_i$ and $y_i$, respectively. M is the number of time points. In the ARACNE algorithm, after calculating pairwise mutual information between all TFs and their targets. Data Processing Inequality (DPI) methods were applied to remove indirect interactions in the network (Margolin et al., 2006).

#### 1.1.2 Random Forest

Random Forest is a tree-based method, which takes into account non-linear relationships between a transcription factor and gene modules (Huynh-Thu et al., 2010). We applied the GENIE3 package, which implemented Random Forest learning method. The goal of this method is to minimize the prediction error in the following equation:

$$Loss = \sum_i (y_i - f(x_{ij}))^2$$

In this equation, $x_{ij}$ and $y_i$ represent expression of TF and target modules, respectively. The subscript $j$ represents all the regulators of a target module. Random Forest algorithm was used to generate 1000 trees for each module to learn the function $f(x_{ij})$ such that the Loss function shown above is minimized (Huynh-Thu et al., 2010). The function $f(x_{ij})$ that minimizes the Loss function represents the best solution that predicts gene expression from regulatory genes.

### 1.1.3 LARS

LARS is a regression-based method with model selection that selects best predictors from a large number of candidate regulators (Haury et al., 2012). LARS uses linear model to represent gene regulatory network with the following equation:

$$y_i = \sum_j \beta_j x_{ij} + \varepsilon$$

The $x_{ij}$ and $y_i$ are the same as in the Random Forest method. The coefficient $\beta_j$ represents the regression coefficient of each regulator and the $\varepsilon$ is an error term. LARS uses a forward, stepwise method to find regulatory TFs that have the strongest effects on the expression pattern of target modules.

### 1.1.4 Partial correlation

The partial correlation method is related to correlation but utilizes a robust estimation method for covariance matrix with small sample sizes (Schafer and Strimmer, 2005a). The partial correlation between a TF ($x_i$) and a target module ($y_i$) is calculated as:

$$PCOR_{ij} = -\frac{\omega_{ij}}{\sqrt{\omega_{ii} * \omega_{jj}}}$$

where $\omega_{ij}$ are the entries of the inversion of the correlation coefficient matrix.

### 1.1.5 CLR method

The CLR method is also based on mutual information; however, CLR incorporates a reweighted Z-score, which takes into account the variable distribution of mutual information between different regulators and gene modules based on the following equation:

$$f(Z_i, Z_j) = \sqrt{Z_i^2 + Z_j^2}$$

In this equation, $Z_i$ is the Z-score of the mutual information between gene $i$ and all other genes $j(M_{ij})$. Similarly, $Z_j$ is the Z-score of the mutual information between gene $j$ and all other genes $i$. Intuitively, for a matrix of mutual information $(M_{ij})$, $Z_i$ is the Z-score for each row whereas $Z_j$ is the Z-score for each column.

## 1.2 Network inference validation

To compare the inferred regulatory interactions between soybean and Arabidopsis, we downloaded three recently published genome-scale regulatory networks from Arabidopsis and merged these data sets (Sparks et al., 2016; Taylor-Teeples et al., 2015; Jin et al., 2015). This merged data set includes 2914 regulatory interactions between 578 regulators and 717 targets. Annotated protein-coding gene sequences for Arabidopsis and soybean were downloaded from TAIR[1] and SoyBase[1], respectively. NCBI BLAST was applied to map each soybean gene to its homologous genes (E-value < 1e-5) in the Arabidopsis proteome. For each TF-module interaction predicted by our methods, we first expanded the TF-module interaction to TF-gene interactions by creating a list of TF-gene interactions between each TF and all genes in the target module. We then searched the

---

[1] http://www.arabidopsis.org/

Arabidopsis interaction network to identify interaction pairs where both TF and the target gene have conserved Arabidopsis homologs in the interaction network database. The predicted interactions and the supporting interaction pairs from the Arabidopsis gene regulatory network database are provided as **Supplementary Table 7**.

To further validate the regulatory interactions, we performed motif search using MEME suite (Bailey et al., 2009). Promoter regions of genes in each module were selected as 1000 bps upstream of transcription start sites of each gene (Liu et al., 2014). All promoter regions were subjected to a motif search using the MEME program with default parameters, except for the width of the motifs, which were set to be between 6 and 12 nucleotides. To define statistically significant enriched motifs, each motif is used to scan promoter regions for all genes used in the clustering analysis using FIMO program. Motif enrichment is tested using Fisher's exact test and the resulting p values are adjusted using the BH method[2]. For all the enriched motifs, we compared these motifs to a recently published large-scale TF binding specificity data set (DAP-seq) (O'Malley et al., 2016) using the TOMTOM program. Significantly similar motifs were selected with p value < 0.01.

### 1.3 Statistical analysis of inference network connections and community-based re-ranking

To estimate the statistical significance of inferred regulatory interactions, we generated expression matrices from the original data and estimated the null distribution of edge weights from permutated data. For example, in the CLR method, CLR calculated a CLR score for each putative regulatory edge in the network. We generated 1,000 permutated expression matrices, and for each permutation, we randomly selected 10,000 interactions and calculated the median of corresponding CLR scores for each network inference method. The 10,000 CLR scores were used to approximate the null distribution of the CLR score and this null distribution was fitted to a Gaussian distribution. For the CLR scores calculated from the original expression data, p-values were estimated using the null distribution. BH method was used to generate FDR adjusted p-value for each edge. Significant edges were selected with FDR adjusted p-value < 0.01. We only compute edge weights from transcription factors to gene modules, because transcription factors are potential regulators whereas gene modules are groups of genes that are potential targets of one or few transcription factors. This method was applied to CLR, ARACNE, PCOR, and Random Forest methods. For the LARS method, although a p-value or FDR was not provided for selecting significant regulations, it provides the ranks of the top 20 regulators. Other putative regulators are regarded as insignificant and are not included in the analysis. We use the rank from LARS method for downstream analysis.

DREAM challenge suggested that a "community-based" approach outperforms individual methods in bench marking experiments. To implement this method, average ranks from each method were used as the final rank of predicted interactions in the inferred network. We implemented this method through two steps: first, statistically significant interactions were selected, and second, ranks for the predicted interactions were averaged as the score for each interaction. In the case where only four out of five methods can predict certain interactions, the method that cannot predict the interaction will be assigned with a minimum rank. We found 46 edges supported by five methods and 341 edges supported by four out of five methods. The inferred network is analyzed to identify edges that start from significantly changed transcription factors. The edges were categorized into five groups: 1) TFs are significantly changed in all four genotypes; 2) TFs are significantly changed only

---

[2] http://www.jstor.org/stable/2346101

in non-mutants (3MWT and 1MWT); 3) TFs are significantly changed only in mutants (*3mlpa* and *1mlpa*); 4) TFs are not significantly changed in any genotype, but changed when comparing between genotypes (3MWT vs. *3mlpa* or 1MWT vs. *1mlpa*); and 5) other types of changes.

## 2 Supplementary Figures and Tables

### 2.1 Supplementary Figures

**Supplementary Figure 1**. **Types of comparisons performed in this analysis.** A) Stage-wise comparison between each pair of matched genotypes (1MWT vs. *1mlpa* and 3MWT vs. *3mlpa*). B) Comparison between adjacent developmental stages for each genotype. S1 through S5 indicate five different developmental stages.

**Supplementary Figure 2. Number of differentially expressed genes in each of the comparisons.**

**Supplementary Figure 3. BIC and number of clusters.**

**Supplementary Figure 4. Predicted regulatory networks.** Grey edges are predicted by 4 methods and red edges are predicted by 5 edges. Orange squares are gene modules. Blue circles are transcription factors. All interactions shown in this figure are provided in supplementary table 4.

### 2.2 Supplementary Tables

**Supplementary Table 1.** Log2 fold change for differentially expressed genes for stage wise comparisons.

**Supplementary Table 2.** Log2 fold change for differentially expressed genes for between stage comparisons.

**Supplementary Table 3.** Log2 fold change for differentially expressed genes with specific function.

**Supplementary Table 4.** Gene Ontology Enrichment Analysis, only included GO terms with p adj < 0.05.

**Supplementary Table 5.** Predicted regulatory interactions.

**Supplementary Table 6.** Predicted regulatory interactions supported by 4 or 5 methods.

**Supplementary Table 7.** Comparing predicted regulation in soybean and known interactions in *Arabidopsis*.

**Supplementary Table 8.** Comparing motifs found in co-regulated gene modules with known *Arabidopsis* motifs.
